# Supplementary material for: Pyruvate Dehydrogenase Kinase 1 inhibition mediated oxidative phosphorylation enhancement in cartilage promotes osteoarthritis progression
Source: BMC Musculoskelet Disord. 2023 Jul 20;24:597. doi: 10.1186/s12891-023-06585-6 (PMC10357736; doi:10.1186/s12891-023-06585-6)
Supplement: Supplementary file 1 — Additional file 1. [file 12891_2023_6585_MOESM1_ESM.zip › BP.Gsea.1653623667859/GOBP_POSITIVE_REGULATION_OF_MITOCHONDRIAL_FISSION.html]

Details for gene set GOBP\_POSITIVE\_REGULATION\_OF\_MITOCHONDRIAL\_FISSION[GSEA]

|  || Dataset | 55457\_collapsed\_to\_symbols.cls.cls #OA\_versus\_normal.cls.cls #OA\_versus\_normal\_repos |
| Phenotype | cls.cls#OA\_versus\_normal\_repos |
| Upregulated in class | OA |
| GeneSet | GOBP\_POSITIVE\_REGULATION\_OF\_MITOCHONDRIAL\_FISSION |
| Enrichment Score (ES) | 0.51604575 |
| Normalized Enrichment Score (NES) | 1.5895498 |
| Nominal p-value | 0.0247191 |
| FDR q-value | 0.2703805 |
| FWER p-Value | 1.0 |
Table: GSEA Results Summary

  

Fig 1: Enrichment plot: GOBP\_POSITIVE\_REGULATION\_OF\_MITOCHONDRIAL\_FISSION      
 Profile of the Running ES Score & Positions of GeneSet Members on the Rank Ordered List

  

| SYMBOL | TITLE | RANK IN GENE LIST | RANK METRIC SCORE | RUNNING ES | CORE ENRICHMENT || 1 | RALBP1 | ralA binding protein 1 [Source:HGNC Symbol;Acc:HGNC:9841] | 217 | 0.808 | 0.1090 | Yes |
| 2 | FIS1 | "fission, mitochondrial 1 [Source:HGNC Symbol;Acc:HGNC:21689]" | 250 | 0.785 | 0.2289 | Yes |
| 3 | MFF | mitochondrial fission factor [Source:HGNC Symbol;Acc:HGNC:24858] | 932 | 0.531 | 0.2589 | Yes |
| 4 | MIEF1 | mitochondrial elongation factor 1 [Source:HGNC Symbol;Acc:HGNC:25979] | 1531 | 0.423 | 0.2785 | Yes |
| 5 | MUL1 | mitochondrial E3 ubiquitin protein ligase 1 [Source:HGNC Symbol;Acc:HGNC:25762] | 1610 | 0.412 | 0.3366 | Yes |
| 6 | PRKN | parkin RBR E3 ubiquitin protein ligase [Source:HGNC Symbol;Acc:HGNC:8607] | 1972 | 0.364 | 0.3653 | Yes |
| 7 | VPS35 | VPS35 retromer complex component [Source:HGNC Symbol;Acc:HGNC:13487] | 2008 | 0.360 | 0.4187 | Yes |
| 8 | PINK1 | PTEN induced kinase 1 [Source:HGNC Symbol;Acc:HGNC:14581] | 2097 | 0.350 | 0.4664 | Yes |
| 9 | MARCHF5 | membrane associated ring-CH-type finger 5 [Source:HGNC Symbol;Acc:HGNC:26025] | 2150 | 0.344 | 0.5160 | Yes |
| 10 | AURKA | aurora kinase A [Source:HGNC Symbol;Acc:HGNC:11393] | 4916 | 0.079 | 0.3137 | No |
| 11 | BNIP3 | BCL2 interacting protein 3 [Source:HGNC Symbol;Acc:HGNC:1084] | 5501 | 0.032 | 0.2734 | No |
| 12 | DDHD2 | DDHD domain containing 2 [Source:HGNC Symbol;Acc:HGNC:29106] | 6517 | -0.043 | 0.2014 | No |
| 13 | DNM1L | dynamin 1 like [Source:HGNC Symbol;Acc:HGNC:2973] | 8499 | -0.194 | 0.0779 | No |
| 14 | DCN | decorin [Source:HGNC Symbol;Acc:HGNC:2705] | 11402 | -0.487 | -0.0714 | No |
| 15 | KDR | kinase insert domain receptor [Source:HGNC Symbol;Acc:HGNC:6307] | 11454 | -0.496 | 0.0020 | No |
| 16 | RALA | RAS like proto-oncogene A [Source:HGNC Symbol;Acc:HGNC:9839] | 12398 | -0.707 | 0.0390 | No |
Table: GSEA details [plain text format]

  

Fig 2: GOBP\_POSITIVE\_REGULATION\_OF\_MITOCHONDRIAL\_FISSION      
 Blue-Pink O' Gram in the Space of the Analyzed GeneSet

  

Fig 3: GOBP\_POSITIVE\_REGULATION\_OF\_MITOCHONDRIAL\_FISSION: Random ES distribution      
 Gene set null distribution of ES for **GOBP\_POSITIVE\_REGULATION\_OF\_MITOCHONDRIAL\_FISSION**
